# Effects of Natural Antioxidants on Phospholipid and Ceramide Profiles of 3D-Cultured Skin Fibroblasts Exposed to UVA or UVB Radiation

**DOI:** 10.3390/antiox10040578

**Published:** 2021-04-08

**Authors:** Agnieszka Gęgotek, Wojciech Łuczaj, Elżbieta Skrzydlewska

**Affiliations:** Department of Analytical Chemistry, Medical University of Bialystok, Mickiewicza 2D, 15-222 Bialystok, Poland; wojciech.luczaj@umb.edu.pl (W.Ł.); elzbieta.skrzydlewska@umb.edu.pl (E.S.)

**Keywords:** skin fibroblasts, rutin, ascorbic acid, UV radiation, phospholipids, ceramides, three-dimensional cell culture

## Abstract

Ultraviolet (UV) radiation is one of the primary factors responsible for disturbances in human skin cells phospholipid metabolism. Natural compounds that are commonly used to protect skin, due to their lipophilic or hydrophilic nature, show only a narrow range of cytoprotective activity, which prompts research on their combined application. Therefore, the aim of this study was to examine the effect of ascorbic acid and rutin on the phospholipid and ceramide profiles in UV-irradiated fibroblasts cultured in a three-dimensional system that approximates the culture conditions to the dermis. An ultra-high-performance liquid chromatograph coupled with a quadrupole time-of-flight mass spectrometer was used for phospholipid and ceramide profiling. As a result of UVA and UVB cells irradiation, upregulation of phosphatidylcholines, ceramides, and downregulation of sphingomyelins were observed, while treatment with ascorbic acid and rutin of UVA/UVB-irradiated fibroblast promoted these changes to provide cells a stronger response to stress. Moreover, an upregulation of phosphatidylserines in cells exposed to UVB and treated with both antioxidants suggests the stimulation of UV-damaged cells apoptosis. Our findings provide new insight into action of rutin and ascorbic acid on regulation of phospholipid metabolism, which improves dermis fibroblast membrane properties.

## 1. Introduction

Human skin—which is responsible for isolation from, as well as communication with, the surrounding environment—ensures the proper functioning of the whole organism. Its multilayer structure, consisting of the epidermis, the dermis, and subcutaneous tissue, provides an effective barrier against external factors such as radiation, xenobiotics, or pathogenic microorganisms [1]. These actions are possible due to mutual interactions between epidermal keratinocytes and dermal fibroblasts [2]. There are a lot of examples of cross-talk between skin fibroblasts and keratinocytes, including not only their bilateral protection against mechanical, chemical and physical factors, but also intercellular signaling [3]. Moreover, skin intracellular composition rich in proteins and numerous lipid derivatives, produced mainly by dermal fibroblasts, protects the organism against water loss or thermoregulation disorders [4]. Therefore, the exposure of fibroblasts to harmful environmental factors, including UV radiation, significantly affects their metabolism and, consequently, the healthy functioning of the skin and the whole organism [5,6].

**Figure 1:**
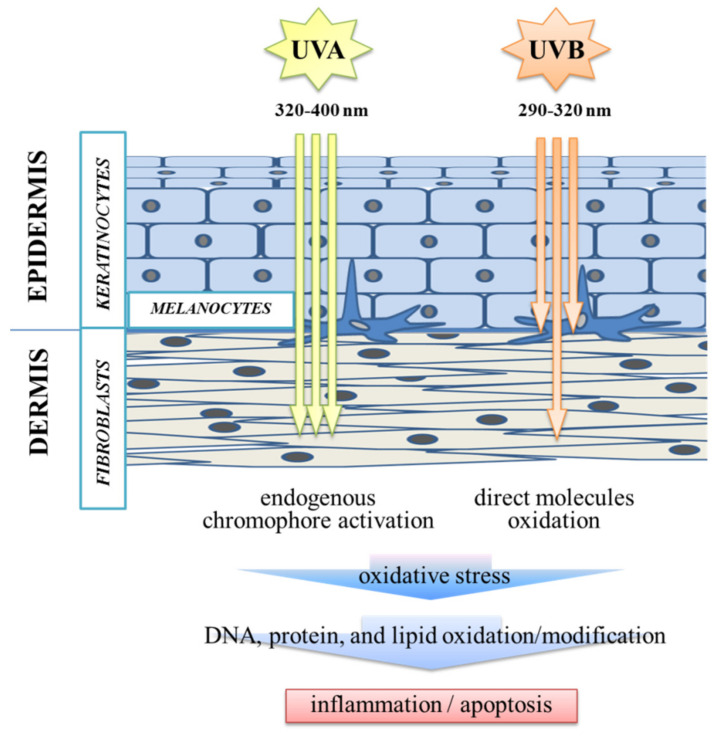
The comparison of the penetration and effects of UVA (320–400 nm) and UVB (290–320 nm) radiation in relation to cells of different skin layers.

The UV radiation type which reaches skin fibroblasts to the greatest extent is UVA, which easily penetrates to the inner layers of the dermis. The next most penetrating bandwidth is UVB, which is mainly absorbed in the epidermis and affects the dermal fibroblasts in only small amounts (Figure 1) [7]. These bandwidths differ not only in wavelength and degree of penetration, but also in the way they affect cellular metabolism. Both bandwidths stimulate the production of reactive oxygen species (ROS). UVA does this by endogenous chromophore activation, while UVB acts directly on molecules to create ROS, the production of which leads to DNA, protein, and lipid damage [8].

However, in both cases, the oxidative stress also induces proinflammatory and proapoptotic signals [5]. This effect, due to the penetration depth connected with the type of radiation in a different way affects the interactions between the cells that build the skin. It has been shown that keratinocytes from epidermis under stress conditions induce the production of proinflammatory and growth factors by dermal fibroblast [3]. This reaction is accompanied by feedback from fibroblasts to keratinocytes hyperproliferation, adhesion, and keratin overexpression [3]. In addition, UVB strongly absorbed by keratinocytes, induces these cell interactions with fibroblasts leading to the epidermal growth factor (EGF) increased secretion [9]. Moreover, molecules involved in signal transduction between fibroblasts and keratinocytes are often products of phospholipid fatty acid metabolism, such as lipid peroxidation products including reactive aldehydes, but also products of enzymatic metabolism such as eicosanoids, endocannabinoids and ceramides [10]. Disturbances in their physiological levels under UV-induced oxidative stress have been identified as a main cause of disruption to skin cell metabolism, thus lowering the functionality of the whole skin [11]. In this connection, there is still a need to identify natural compounds with skin-protective properties, especially with regard to cellular phospholipids.

To date, many cytoprotective compounds have been identified with antioxidant effects; however, most of them have a lipophilic or hydrophilic character which means they can only act in one fraction: membrane or cytosol [12]. Therefore, systems of compounds that would protect both cellular fractions are sought. A good solution seems to be the use of two well-known antioxidants—the water-soluble ascorbic acid and the partially lipophilic rutin—which both exhibit antioxidant properties and independently protect cell metabolism [13,14]. So far, these compounds have been used together with great success in oral pharmaceutical preparations, increasing the body’s immunity [15,16].

The biochemical functions of ascorbic acid include stimulation of the antioxidant system; activation of certain enzymes; collagen biosynthesis, hormonal activation; detoxification of histamine; proline hydroxylation [17]. Rutin is known to modulate the permeability of the blood vessels and to support the antioxidant system as a free radical scavenger, as well as being an activator of the transcription factor Nrf2 [14,18].

Moreover, our previous studies show that ascorbic acid and rutin used together have a synergistic effect in stimulation of the cellular antioxidant system and protection against changes to the proteomic profile of UV-irradiated keratinocytes and fibroblasts [19,20]. Moreover, in the case of skin fibroblasts, these properties become even more pronounced if the traditional single-layer cell culture model is changed to a three-dimensional (3D) model [21,22]. Since fibroblasts in the dermis are constantly surrounded by other cells, observations made under 3D model conditions are more representative of physiological conditions. Exposure to UV radiation will elicit a response, that depends not only on the type of radiation, but also on the degree of ray penetration into the cell’s physiological environment, which cannot be assessed in the case of single-layer cell culture. The same is true in the case of cell treatments with chemical factors, where 3D modeling allows the discovery of more representative intra- and extracellular signaling pathways as compared to single-layer culture [23,24]. The multilayer structure of the dermis, combined with the action of the cell membrane, is a strong barrier to be overcome for compounds with a potential protective effect. In the case of rutin its action is limited due to its retention on skin surface and keratinized layers, that is equal to 95% of total topically applied dose [25], while ascorbic acid permeation through skin model varies even in range 20–40% [26]. Therefore, in the case of ascorbic acid/rutin cooperation, ascorbic acid is beneficial in that it facilitates the penetration of rutin into the cell [27]. This, combined with the proteome preservation mentioned above, suggests that these compounds could protect lipids and their derivatives, and therefore are extremely important in the functioning of the skin, especially under stress conditions.

Therefore, the aim of this study was to analyze and compare the effects of ascorbic acid and rutin, when used either separately or together, on the phospholipid and ceramide profiles of 3D-cultured skin fibroblasts following UVA or UVB radiation.

## 2. Materials and Methods

### 2.1. Reagents/Chemicals

Human skin fibroblasts (CRL-1474) were obtained from the American Type Culture Collection (ATCC). Sterile reagents for cell culture, including AlgiMatrix 3D Cell Culture System, were obtained from Thermo Fisher Scientific (Waltham, MA, USA). Phospholipid and ceramide internal standards were purchased from Avanti Polar Lipids, Inc. (Alabaster, AL, USA). All chemicals were purchased from Sigma-Aldrich Chemical Co. (St. Louis, MO, USA). All solvents were of LC-MS grade. Milli-Q water was used for all experiments, filtered through a 0.22 µm filter and obtained using a Milli-Q Millipore system (Advantage A10, Millipore Corporation, Billerica, MA, USA).

### 2.2. Cell Culture

Prior to transfer to a 3D substrate, cells were cultured in a two-dimensional model in a humidified atmosphere of 5% CO_2_ at 37 °C in a growing medium consisting of Dulbecco’s Modified Eagle Medium (DMEM) supplemented with 10% fetal bovine serum (FBS) and 50 μg/mL streptomycin and 50 U/mL penicillin. When cells (passage no. 9) reached 90% confluence, they were seeded into 24-well plates (5 × 10^5^ cells/well) with AlgiMatrix gel to create a three-dimensional model, and cultured under standard conditions in a growing medium (DMEM, 10% FBS, 50 μg/mL streptomycin and 50 U/mL penicillin) for four days, according to the producers protocol [28]. Next, cells were washed with phosphate-buffered saline (PBS) and in this buffer exposed to UVA (365 nm) at a dose of 20 J/cm^2^, or to UVB (312 nm) at a dose of 200 mJ/cm^2^, using a Bio-Link Crosslinker BLX 365/312 (Vilber Lourmat, Germany). UV doses were chosen to reflect 70% cell viability in a 2D culture [13]. 

**Figure 2:**
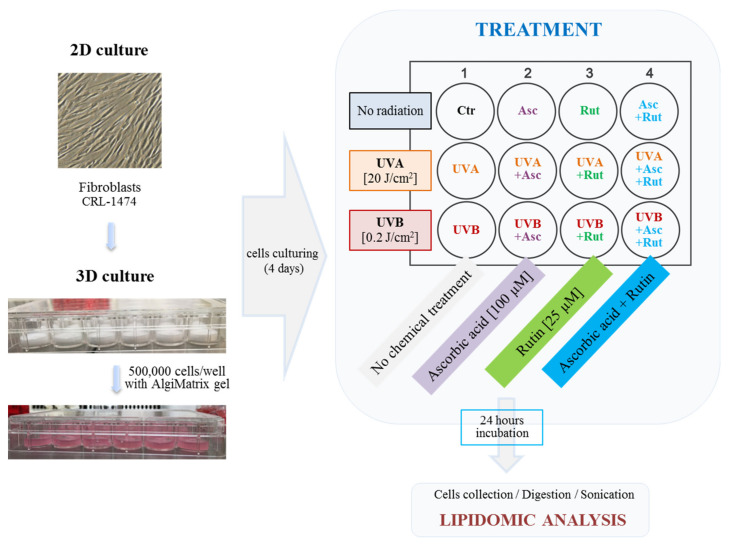
The overview of the experiment: cell culture preparing and tested groups. Abbreviations: Asc, ascorbic acid; Rut, rutin.

Following irradiation, control group cells were cultured in a fresh medium without FBS under standard conditions for 24 h. To analyze the effect of ascorbic acid and rutin on UV-irradiated cells, cells were then incubated for 24 h in medium containing either 100 µM ascorbic acid [13] and/or 25 µM rutin in 0.1% DMSO [29]. Concentrations were selected according to the cells viability measured by the sulforhodamine B assay in a 2D culture, doses that were used did not reduce cell survival, but at the same time were sufficient to cause an effect on cells metabolism, including activation of the antioxidant enzymes or Nrf2 pathway, as well as reduction the lipid peroxidation products level [19]. Control cells were cultured in parallel with no treatment. Following incubation, fibroblasts were recovered from the 3D gel with the use of AlgiMatrix™ dissolving buffer, then lysed by sonication on ice, and centrifuged (15 min, 12,000× g). The creation of a cell culture for the experiment, as well as cells treatments are shown in Figure 2.

### 2.3. Lipidomic Analysis

#### 2.3.1. Extraction of Lipids and Total Phospholipid Quantification 

Total lipids were extracted from cell pellet with the use of the Bligh and Dyer method [30]. Quantification of the amount of phospholipids in each extract was performed according to the Bartlett and Lewis method [31]. Experimental procedures of lipid extraction and phospholipid quantification were described in detail in a previously published study based on the same methodology [32].

#### 2.3.2. Phospholipid Separation and Quantification by UPLC-ESI-MS

Hydrophilic interaction liquid chromatography (HILIC) was applied to separate phospholipids with the use of a ultra-high performance liquid chromatography (UPLC) system (Agilent 1290; Agilent Technologies, Santa Clara, CA, USA) coupled to a quadrupole time-of-flight (QTOF) mass spectrometer (Agilent 6540; Agilent Technologies, Santa Clara, CA, USA). In order to confirm and quantify the ion variations the phospholipid standards dMPC (PC 14:0/14:0), LPC (19:0), dMPE (PE 14:0/14:0); CL (14:0/14:0/14:0/14:0), dPPI (PI16:0/16:0), and dMPS (PS 14:0/14:0) were used. The solvent system was composed of mobile phase A (acetonitrile, methanol and water with 1 mM ammonium acetate, 50%, 25% and 25% (*v*/*v*)) and mobile phase B (acetonitrile and methanol with 1 mM ammonium acetate, 55% and 45% (*v*/*v*)). The solvent gradient was initiated with 0% mobile phase A, increasing linearly to 100% within 20 min, holding for 15 min, and then returning to initial composition of mobile phase in 10 min. The mobile phase flow rate was 40 μL min^−1^.

In total, 25 μg of lipid extract of each sample diluted in phospholipid standards mixture and eluent B, was loaded onto the chromatographic column (Ascentis^®^ Si, 15 cm × 1 mm; 3 μm, Sigma-Aldrich). All MS analyses were carried out in negative-ion mode with electrospray voltage, −3000 V; sheath gas flow, 13 L/min; capillary temperature, 250 °C, as typical ESI conditions. Data-dependent acquisition mode (DDA) was used for data collecting. Parent ion scanning was performed in the *m*/*z* range of 100–1500, while the collision energy was setup at 35 eV. The LPC, PC and SM species were analyzed as [M + CH_3_COO]^−^ adducts, while other phospholipid species were analyzed as [M − H]^−^ ions. The Agilent Mass Hunter data software (version B0.8.0) was used for data acquisition. Relative abundance of each ion was estimated by normalization to the peak area of an internal standard. Phospholipid species identification was based on the retention times and inspection of the MS/MS spectra.

#### 2.3.3. RPLC-ESI-MS Analysis 

Reversed-phase (RP) chromatography LC-MS/MS was utilized to characterize ceramide (CER) profiles. The same UPLC-ESI-QTOF-MS system (Agilent 1290; Agilent 6540; Agilent Technologies, Santa Clara, CA, USA) was used for the analysis. The separation of ceramides was carried out on an RP C18 column (Acquity BEH Shield 2.1 × 100 mm; 1.7 μm; Waters, Milford, MA, USA). The mobile phase consisted of water with 20 mM ammonium formate at pH 5 (A) or methanol (B). The solvent gradient started at 70% eluent B held for 1 min, linearly increasing to 100% within 75 min, and returning to initial composition over a final 5 min period. Flow rate was 0.5 mL/min. The MS analysis was performed in positive-ion mode. Electrospray voltage set to 3.5 kV; the drying and sheath gas temperatures set to 300 °C, and the drying and sheath gas flow rates set to 6 and 8 L/min, respectively, were the typical ESI conditions. Data was acquired in DDA mode. Ceramides were identified according to the presence of the [M + H]^+^ molecular ion, retention time, and characteristic fragmentation patterns, as described previously [33].

#### 2.3.4. Data Processing

The assignment of each phospholipid and ceramide species was performed with the use of MZmine software version 2.30 [34]. The software enables also filtering, peak detection, alignment, and integration. 

#### 2.3.5. Statistical Analysis

Data are presented as mean ± standard deviation. Metaboanalyst version 4.0 was used for the univariate and multivariate statistical analyses [35]. The obtained by MS/MS analysis data were autoscaled and subjected to principal component analysis (PCA). The processed data was analyzed for statistical significance using a one-way ANOVA with an adjusted *p*-value (FDR) cutoff of 0.05, and Tukey’s post-hoc tests. The heat map was constructed with the use of “Euclidean” clustering distance and “Ward” clustering algorithm.

## 3. Results

In this work, we used a high-resolution HILIC-LC-MS/MS platform to characterize the changes in the phospholipid profile of fibroblasts exposed to UVA or UVB radiation and treated with rutin and/or ascorbic acid. We also used an RPLC-QTOF-MS/MS platform to evaluate alterations to the ceramide profile. We identified phospholipid species belonging to phosphatidylcholine (PC), lyso-PC (LPC), phosphatidylethanolamine (PE), lyso-PE (LPE), phosphatidylinositol (PI), phosphatidylserine (PS), and sphingomyelin (SM) classes. The list of 104 phospholipid species (corresponding to the most abundant species each of their respective classes) which were identified and quantified is presented in Supplementary Table S1. For the relative quantification of all phospholipids listed in Supplementary Table S1, the peak areas of the extracted ion chromatograms of each PL species within each class were normalized using the peak area of the internal standard (ISTD) selected for the class. 

**Figure 3:**
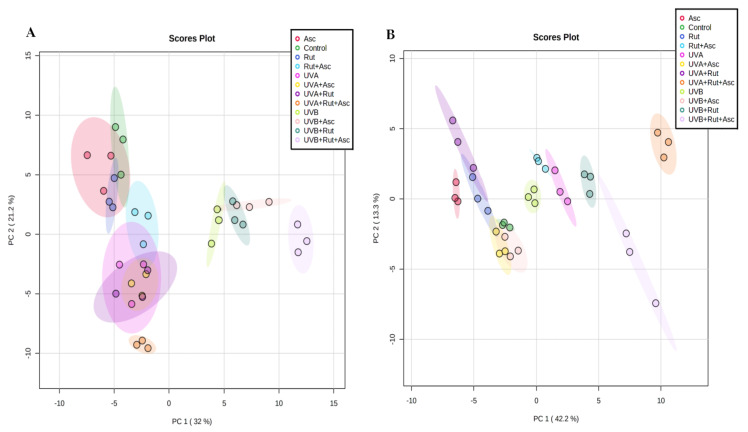
Two-dimensional principal component analysis (2D PCA) scores plot of the relative phospholipid (**A**) and ceramide (**B**) content related to the internal standard of each class within each class in 3D cultured fibroblasts: not treated (Control); treated with ascorbic acid (Asc); treated with rutin (Rut); treated with rutin and ascorbic acid (Rut+Asc); exposed to UVA (UVA); exposed to UVA and treated with ascorbic acid (UVA+Asc); exposed to UVA and treated with rutin (UVA+Rut); exposed to UVA and treated with rutin and ascorbic acid (UVA+Rut+Asc); exposed to UVB (UVB); exposed to UVB and treated with ascorbic acid (UVB+Asc); exposed to UVB and treated with rutin (UVB+Rut); exposed to UVB and treated with rutin and ascorbic acid (UVB+Rut+Asc).

In the case of ceramide profiles of our 3D-cultured fibroblasts, we identified more than 60 ceramide species, including non-hydroxy fatty acid (N), α-hydroxy fatty acid (A), and esterified ω-hydroxy fatty acid (EO), plus three sphingoid bases (dihydrosphingosine (DS), sphingosine (S), and phytosphingosine (P)). All identified ceramides belong to seven different classes, namely CER[NS], CER[NDS], CER[NP], CER[ADS], CER[AS], CER[AP], and CER[EOS] (Supplementary Table S2). Identification of CER species was based on the presence of the molecular [M + H]^+^ ion, retention time, and typical fragmentation patterns observed in the MS/MS spectra. Multivariate and univariate statistical analyses were used to identify significant changes in the profiles of phospholipids and ceramides between groups. The data were first autoscaled and then subjected to a principal component analysis (PCA) to reveal the clustering trends of the experimental groups (Figure 3).

The two-dimensional principal component analysis (2D PCA) plot corresponding to the phospholipid data set of 12 analyzed groups shows that the model captured 53.2% of the total variance (Figure 3A). The variation between the different biological groups is more pronounced with the PC1 component (32%), which accounts for the greatest variation, and allows discrimination of fibroblasts irradiated with UVB from other groups of analyzed cells. The second component, PC2 (21.2%), mainly accounts for the variation between groups of control fibroblasts and groups of UVA-irradiated cells. The PCA plot shows that these groups (UVA-irradiated cells) as well as control fibroblasts are scattered in the left region of the plot and clearly separated from the groups of fibroblasts irradiated with UVB, which were scattered in the right region of the plot (Figure 3A). Separation was most pronounced between groups of UVB-irradiated cells and the rest of the analyzed groups. Although control cells treated with rutin and/or ascorbic acid were poorly separated from each other, they were discriminated from groups of UVA-irradiated cells by the PC2 component.

The PCA two-dimensional plot constructed for ceramide profiling represented the analyses describing 55.5% of the total variance, including PC1 (42.2%) and PC2 (13.3%), where PC1 was the major discriminating component (Figure 3B). While the samples irradiated with UVA or UVB were scattered in the center of the plot, PC1 mainly accounts for the variation between the cluster of nonirradiated fibroblasts plus those irradiated with UVA/UVB and treated with rutin and ascorbic acid simultaneously, which were scattered in the right region of the plot, versus the rest of the analyzed groups, scattered on the left region. However, PC2 most probably describes the variation between groups of UVA and UVB-irradiated fibroblasts treated with both antioxidants simultaneously.

**Figure 4:**
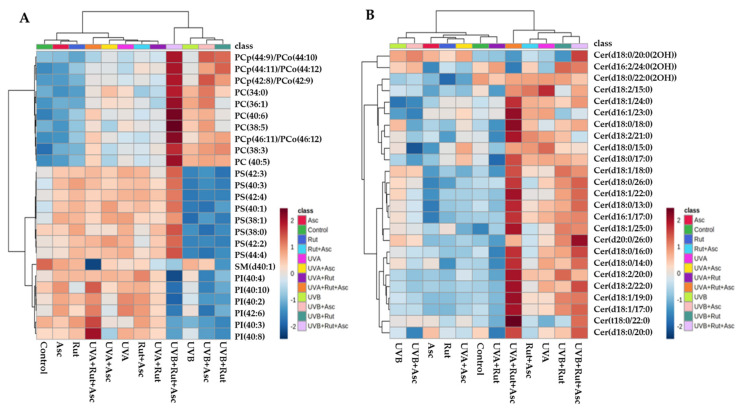
Two-dimensional hierarchical clustering heat map of the 25 most discriminating phospholipid (**A**) and ceramide (**B**) molecular species (according to one-way ANOVA and Tukey’s post-hoc tests) of the 12 studied 3D cultured fibroblasts groups. Levels of relative abundance are indicated on the color scale, with numbers indicating the fold difference from the grand mean. The clustering of the sample groups is represented by the dendrogram on the top. The clustering of individual phospholipid species with respect to their similarity in change of relative abundance is represented by the dendrogram to the left. Abbreviations: Asc, ascorbic acid; Rut, rutin.

Finally, we performed a univariate analysis (one-way ANOVA and Fisher’s LSD post-hoc tests) in order to assess the variation of the relative abundance of the molecular species of phospholipids and ceramides under the conditions studied. The univariate analysis was used to create a dendrogram with two-dimensional hierarchical clustering, using the 25 main phospholipids (Figure 4A) and ceramide (Figure 4B) species, according to one-way ANOVA. The primary split in the upper hierarchical dendrogram shows that the samples were clustered independently in three main groups, in both phospholipid and ceramide analyses (Figure 4A,B). Clustering of the individual lipid species (with regard to their similar expression changes) shows that they cluster into three and four main groups for phospholipids and ceramides, respectively. In the case of phospholipids, the first group was mainly composed of PC species. The second group consisted of PS, while the third group included PI species and one SM species, namely SM(d41:0). The first group of clustered ceramides was composed of α-hydroxylated CER (CER[AS] and CE[ADS]), while the other three clusters were composed of ceramides and dihydroceramides (CER[NS] and CER[NDS]) (Figure 4B).

### 3.1. Comparison of Phospholipid and Ceramide Profiles of Control Fibroblasts vs. Those Treated with Rutin or Ascorbic Acid Separately, as well as with Both Compounds together (Control vs. Rut vs. Asc vs. Rut+Asc)

For a more detailed interpretation of the data, we decided to investigate changes by analyzing three data sets, each comprising four different groups. 

The first set included data from the following groups: control; rutin alone (Rut); ascorbic acid alone (Asc); rutin plus ascorbic acid (Rut+Asc). Among the phospholipid profiles of these groups, we found that PC species were upregulated in the fibroblasts treated with Rut and Asc—especially PC(40:6), PC(38:5), PC(38:3), PC(40:5), and PC plasmalogen PCp(46:11). Moreover, a general tendency towards a decrease in SM(d40:1) relative content was shown after Rut or Asc treatment. However, when compared to untreated cells, the most significant downregulation of SM(d40:1) was observed in fibroblasts treated with both Rut and Asc (Figure 4A, Table 1, Supplementary Table S3), which was accompanied by a significant upregulation of ceramide species (mainly Cer(d18:2/15:0); Cer(d18:1/24:0); Cer(d16:1/23:0); Cer(d18:0/18:0); Cer(d18:2/21:0); Cer(d18:0/15:0); Cer(d18:0/17:0) (Figure 4B, Table 2, Supplementary Table S4)). 

In contrast, cells treated only with Asc showed a tendency towards downregulation of ceramides, including some CER[NS] and CER[NDS] species (Cer(d18:0/15:0); Cer(d18:0/17:0); Cer(d18:1/18:0); Cer(d18:0/26:0); Cer(d18:1/22:0); Cer(d18:0/13:0); and Cer(d16:1/17:0)). Interestingly, in comparison to control cells, significant changes were observed in α-hydroxylated ceramides in fibroblasts treated with Rut or Asc—namely, upregulation of Cer(d18:0/22:0(2OH)), and downregulation of both Cer(d16:2/24:0(2OH)) and Cer(d18:0/22:0(2OH)). 

Significant changes in phospholipid and ceramide profiles were also observed in fibroblasts irradiated with UVA or UVB. These changes include upregulation of PC species and downregulation of sphingomyelin SM(d40:1) in both groups of fibroblasts irradiated with UVA and UVB (Figure 4A, Table 1, Supplementary Table S3). Moreover, a general tendency towards increase of CER species was observed in fibroblasts irradiated with both types of UV light, but more pronounced upregulation was noted for cells exposed to UVA, in which almost all relevant CER species were upregulated (Figure 4B, Table 2, Supplementary Table S4). However, we found interesting changes in the relative abundance of phosphatidylserine (PS) and phosphatidylinositol (PI) species. Exposure of fibroblasts to UVA radiation led to upregulation of all significantly relevant PS and PI species, with the exception of PI(40:3). In contrast, UVB led to significant downregulation of both classes.

### 3.2. Comparison of the Phospholipid and Ceramide Profiles of Fibroblasts Exposed to UVA and then either Not Treated, Treated Separately with Rutin or Ascorbic Acid, or Treated with Both Compounds Together (UVA vs. UVA+Rut/UVA+Asc/UVA+Rut+Asc)

To examine changes in phospholipid and ceramide profiles of UVA-irradiated fibroblasts resulting from treatment with rutin or ascorbic acid, we analyzed a second set of data composed of the following groups: UVA, UVA+Rut, UVA+Asc, and UVA+Rut+Asc. We found no significant differences between the phospholipid profiles of UVA-irradiated fibroblasts after ascorbic acid or rutin treatment, with the exception of a slight downregulation of some PI species (namely PI(40:2), PI(42:6), and PI(40:8) (Figure 4A, Table 1, Supplementary Table S3)). It should be underlined that in comparison to untreated UVA-irradiated cells, exposure of these cells to both compounds simultaneously (Rut+Asc) led to a dramatic downregulation of sphingomyelin species SM(d40:1) (Figure 4A, Table 1, Supplementary Table S3). The decrease in relative content of this SM species was accompanied by upregulation of almost all relevant CER species, which was most significant among all experimental groups examined in the present study (Figure 4B, Table 2, Supplementary Table S4). 

Interestingly, treatment of UV-irradiated fibroblasts with either Rut or Asc alone resulted in significant downregulation of most ceramides (Figure 4B). In addition to these changes, when compared with the other groups of UVA-irradiated fibroblasts, we also noted significant upregulation of PI species, especially PI(40:3) and PI(40:8) in UVA-irradiated cells treated with Rut and Asc.

### 3.3. Comparison of the Phospholipid and Ceramide Profile of Fibroblasts Exposed to UVB and then either Not Treated, Treated Separately with Rutin or Ascorbic Acid, or Treated with Both Compounds Together (UVB vs. UVB+Rut vs. UVB+Asc vs. UVB+Rut+Asc)

Assessment of the third set of data (UVB, UVB+Rut, UVB+Asc, and UVB+Rut+Asc) revealed the most pronounced changes in the phospholipid profile of fibroblasts irradiated with UVB and treated with rutin and ascorbic acid simultaneously. We found significant upregulation of all statistically relevant PS species (Figure 4A, Table 2, Supplementary Table S4)—namely PS(42:3), PS(40:3), PS(42:4), PS(40:1), PS(38:1), PS(38:0), PS(42:2), and PS(44:4). In addition, a dramatic increase was observed in the relative abundance of all PC and PCp species in this group of cells (Figure 4A, Table 1, Supplementary Table S4). We also observed a significant decrease in the relative abundance of SM(d40:1) after treatment of UVB-irradiated fibroblasts with rutin alone, and this was even more pronounced when these cells were treated together with rutin and ascorbic acid together. These observations were correlated with an upregulation of most of ceramide species indicated in fibroblasts exposed to UVB and treated with rutin, but especially when treated with rutin and ascorbic acid simultaneously. We also found significant downregulation of most PI species in UVB-irradiated cells after rutin and/or ascorbic acid treatment when compared to UVB-irradiated cells without treatment (Figure 4A, Table 1, Supplementary Table S3).

## 4. Discussion

UV radiation as one of the most harmful factors reaching human skin in various ways affects cell metabolism depending both on the type of UV radiation and the skin cell line subject to irradiation [5,36]. However, these UV-induced changes are strongly dependent also on cell-to-cell interactions, based on signaling molecules secretion [37]. It has been described that epidermal keratinocytes, creating the first line of protection against e.g., UV radiation, transmit information about this factor, as well as about its negative effects into skin fibroblasts. As a result, increased cells proliferation or even differentiation are observed, which can lead to the development of skin cancer [38,39,40]. On the other hand, 3D cocultured fibroblasts with keratinocytes were shown as proapoptotic signal inducers causing increase in caspase-3 and Bad expression in keratinocytes [41]. That shows how important the interactions between UV irradiated skin cells are, as well as the level of signaling molecules they produce, especially lipids and ceramides.

Phospholipids and ceramides represent the main lipid species present in skin cells that are not only responsible for organization of membrane structure, but which also play an important role in cell signaling, as well as the permeability of cell membranes. It is well-known that UV radiation, particularly by the induction of oxidative stress, leads to the alteration of skin phospholipid and ceramide metabolism, and consequent changes in their levels and composition [42]. Until now, most research has focused on the effect of UV radiation on the phospholipid metabolism in major epidermal cells—keratinocytes [32,43,44]. Unfortunately, these studies do not reflect the metabolic consequences of UV radiation in the dermis, the deeper layer of the skin, the primary cells of which are the fibroblasts. Through the release of cytokines and growth factors, fibroblasts play a crucial role in regulating various processes in the skin, including signal transduction which facilitates their interaction with the surrounding extracellular matrix and blood vessels [45]. 

The results obtained in this study show that both UVA and UVB induce changes in the 3D-cultured fibroblast phospholipid profile, namely the upregulation of PC species and downregulation of sphingomyelin SM(d40:1), which has also been shown in our previous in vitro studies on fibroblasts cultured in a 2D system [46]. Downregulation of sphingomyelin species SM(d40:1) was accompanied by a general tendency to increase the level of ceramides belonging to CER[NS] (ceramides containing non-hydroxy fatty acids and sphingosine) and CER[NDS] (ceramides containing non-hydroxy fatty acids and dihydrosphingosine). Such increased ceramide synthesis in the epidermis following UV exposure has been reported previously [47,48]. In addition, it has also been reported that UV radiation accelerates the formation of ceramides through hydrolysis of sphingomyelin by increasing acidic-sphingomyelinase activity in human dermal fibroblasts [49]. Moreover, it has also been shown that UV radiation by causing oxidative stress increases the expression of acid and neutral sphingomyelinases at the mRNA level [50]. This is in agreement with the results presented above, which may indicate the stimulation of sphingomyelin/ceramide pathways, since the enzymatic hydrolysis of sphingomyelins is one of the main mechanisms leading to formation of ceramides [51]. Our results show more pronounced upregulation of all relevant CER species in fibroblasts exposed to UVA, which may be explained by the ability of UVA radiation to penetrate more deeply into the skin [42]. Moreover, it has also been shown that UVA radiation is also involved in the generation of singlet oxygen, which has been suggested as a nonenzymatic mechanism for ceramide formation [52]. In fact, it has been indicated previously that UVA radiation triggers de novo ceramide synthesis, as well as enhancing enzymatic pathways of ceramide generation in human skin cells through sphingomyelinase action [53,54]. 

The results of this study also reveal inverse changes in the content of phosphatidylserines and phosphatidylinositols, depending on the type of UV light used for irradiation. All relevant PS and PI species were upregulated in UVA-irradiated fibroblasts, while downregulation of these phospholipids was observed in cells exposed to UVB radiation. It may be assumed that phosphatidylserine upregulation may be associated with its translocation to the outer layer of the membrane, which is an important indicator of apoptosis under the influence of ROS [55,56]. However, it may be also suggested that a stronger overproduction of ROS, observed in fibroblasts exposed to UVB rays [5], may favor the oxidation of PS [57,58]. This may explain the reduction in relative PS levels observed in UVB-irradiated fibroblasts. However, since UV radiation activates phosphatidylinositol 3-kinase, it may be suggested that observed downregulation of PI species resulted from their phosphorylation to phosphoinositides (e.g., PI(4)P and PI(4,5)P2) by phosphatidylinositol kinases [59]. It is plausible that these metabolic changes attributable to UV radiation might also be stimulated in phototherapy for skin diseases such as atopic dermatitis or psoriasis [60,61], which emphasizes the importance of developing novel therapies based on UV radiation due to the simple stimulation of these natural cytoprotective compounds. 

Cells in the skin of healthy people are also often exposed to harmful levels of UV radiation from sunlight, e.g., as a result of excessive sunbathing, which means that compounds/preparations are sought to prevent deleterious metabolic changes. One of the consequences of high doses of UV irradiation is redox imbalance. Thus, one desirable characteristic of a protective compound is the possession of antioxidant properties. Two examples of such compounds are ascorbic acid and rutin, which, when used together, exert an antioxidant effect in both the aqueous and lipid environment that has been tested and proven in relation to the proteome of skin cells [19,20]. 

To date, very few reports have been published on the cooperative effect of the compounds mentioned above on lipid metabolism [46,62]. Although no significant changes in phospholipid profile have been indicated in our previous study on fibroblasts cultured in a 2D system [46], the results of this current study show upregulation of phosphatidylcholine species in UVA- and UVB-irradiated cells, both of those that were treated and those which were untreated with ascorbic acid and/or rutin. This may have implications for cell signaling and intercellular transport. Recently, it has been shown that the specific ascorbate phosphatidylcholine (PC) liposome has an ability to overcome the barrier of the stratum corneum and deliver the active agents into the dermis to prevent photodamage [63]. Therefore, the observed upregulation of phosphatidylcholine species, especially in both groups of nonirradiated and UVB-irradiated fibroblasts treated with rutin and ascorbic acid, may be associated with a specific mechanism allowing active compound (e.g., rutin) penetration into the deeper fibroblast layers in a 3D system—or even into the layers of the dermis. It has been shown previously that increase in PC species is pivotal to rutin action since they also facilitate its transport through the cell membrane. Rutin and phosphatidylcholine interact to form a PC-rutin complex known as a phytosome, which can help to overcome difficulties in medicinal fortification, such as low water solubility and bioavailability [64]. Moreover, quite recently it has been proven that the formation of a nano-complex of rutin with phosphatidylcholine provides better efficacy of biological rutin action [65]. Therefore, taking into account the results obtained here, it may be suggested that ascorbic acid, assisted by the antioxidant activity of rutin, increases the synthesis of phosphatidylcholine, consequently facilitating the transport of rutin through the fibroblast layers, but also probably allowing its penetration into the deeper dermis. 

The cooperative action of both antioxidants may also explain a decrease in the relative abundance of SM(d40:1) and also the accompanying significant upregulation of ceramide species (mainly of the CER[NS] and CER[NDS] classes) in fibroblasts irradiated and treated with both antioxidants. In addition, our results indicate the downregulation of fibroblast ceramides by ascorbic acid alone, which stands in contrast to previous findings which suggest that ascorbic acid enhances de novo ceramide synthesis by activation of serine palmitoyltransferase and ceramide synthase, while sphingomyelinase remains unchanged [66]. Notably, those findings were reported in 2D-cultured keratinocytes. This may suggest different changes in the ceramide metabolic pathway in skin and epidermal cells and different responses depending on the 2D or 3D nature of the culture. In addition to indicated downregulation of CER[NS] and CER[NDS] a significant upregulation of hydroxylated ceramide, namely Cer(d18:0/22:0(2OH)) was also observed in all groups of fibroblasts treated with ascorbic acid. It has previously been shown that ascorbic acid facilitates the hydroxylation of sphingoid bases and fatty acids, generating α-hydroxy fatty acids, ω-hydroxy fatty acids, or various sphingoid bases [67]. This may explain the observed increases in relative abundance of Cer(d18:0/22:0(2OH)) in all groups of fibroblasts treated with ascorbic acid. However, our study demonstrated a significant increase of most relevant ceramide species in control fibroblasts, as well as in UVA or UVB-irradiated cells, when exposed to both rutin and ascorbic acid simultaneously. Taking this together with the downregulation of SM(d40:1), these findings may suggest induction of sphingomyelin degradation through sphingomyelinase action. Moreover, the decrease in SM(d40:1) may additionally suggest combined effect of both compounds, which has been reported by earlier metabolic and proteomic studies on skin cells exposed to UVA/B radiation [19,20]. 

Since the most important function of the epidermal barrier is to prevent excessive water loss, and since ceramides are considered the main type of lipid that ensures proper skin permeability, the observed upregulation of ceramides indicates an important role for the combined action of rutin and ascorbate in preventing the alteration of permeability [68]. It has been shown that ceramides derived from sphingomyelinase activation typically accumulate shortly after the stimulus is triggered [69], which may suggest this is a result of the immediate effects of UV, while de novo synthesized ceramides accumulate later. Therefore, it could be suggested that the increase in the level of ceramides after UVA/UVB irradiation may be mainly the result of an increase in existing sphingomyelinase activity, while the increase in relative ceramide content observed after treatment with antioxidants (rutin and ascorbic acid) may be more attributable to de novo synthesis. In addition to these changes, there was a significant upregulation of PI species in UVA-irradiated fibroblasts treated simultaneously with rutin and ascorbic acid, while in UVA-irradiated cells treated only with rutin or ascorbic acid, the general trend was to lower PI levels (as was observed in case of all UVB-irradiated cells). Moreover, in UVB-irradiated fibroblasts treated with both rutin and ascorbic acid, a dramatic upregulation was observed in all relevant PS species. These changes are probably the result of the cumulative antioxidant properties of two natural compounds preventing oxidative modifications of PS by ROS, to which these phospholipids are particularly sensitive [57]. The upregulation of PS may be very important, as indicated by the way these phospholipids bind to PPARα receptors, which reduce UVB-induced inflammation [70]. Therefore, the upregulation of phosphatidylserines resulting from treatment of UVB-irradiated fibroblasts with ascorbic acid and rutin may be a part of the response to inflammatory processes induced by UV radiation. 

Ceramide, by modifying the structure of the membranes, contributes to the change of the mitochondrial membrane potential by specialized proteins such as the Bcl-2 family, which leads to the release of cytochrome C and apoptosis-induced factor (AIF) into the cytoplasm [71]. Moreover, the increased accumulation of ceramide leads to the formation of ceramide-rich domains in the membranes, which provide a platform for the accumulation and activation of cytokine receptors, including TNFR1 and death receptors [72]. Considering that the proapoptotic effect is related to the activation of the executive enzymes—caspase 3 and caspase 8, which degrade the cytoskeleton, biological membranes and the cell nucleus—the increase of ceramide in UVB-irradiated fibroblasts exposed to both ascorbic acid and rutin may suggest the participation of ceramide in proapoptotic signal transduction in these cells. Therefore, the association of phosphatidylserines and ceramides with reduction of inflammation, and with the proapoptotic mechanism of action of ascorbic acid and rutin, may indicate the cooperation of these two antioxidants in a smooth change in the direction of metabolic processes of fibroblasts from proinflammatory responses after UV radiation to degradation of damaged cells by apoptosis.

## 5. Conclusions

In conclusion, our results showed that the combined action of both tested compounds led to significant changes in the profile of phospholipids and ceramides. The results obtained in the study indicate the protective effect of rutin and ascorbic acid, especially after irradiation of fibroblasts with UVA/UVB radiation, which leads to significant changes in the phospholipid and ceramide profiles. These changes include upregulation of phosphatidylcholines and ceramides, as well as downregulation of sphingomyelins, that provide a stronger response of cells to stress. Meanwhile, the application of each antioxidant compound on its own resulted in an opposite change in phospholipid levels. Our findings provide new insight into the role of both compounds acting together to regulate dermal lipid metabolism. In particular, these results suggest mechanisms for these compounds related to apoptosis and the improvement of membrane properties—mainly decrease of permeability and inhibition of intracellular water loss—through increased ceramide generation. In addition, considering obtained results and the multitude of interactions between fibroblasts and keratinocytes, in order to fully understand the mechanisms induced by the action of antioxidants following the exposure to UV radiation, it is also necessary to study the alteration of lipid profile in a common model of cocultures containing keratinocytes and fibroblasts. This may be of interest to many researchers in the field of skin diseases pharmacotherapy, particularly due to the high therapeutic potential of both natural antioxidants examined. Nevertheless, further research is needed to elucidate the exact mechanisms by which both used compounds act, and also to explain the metabolic changes suggested in this study. 

## Figures and Tables

**Table 1:** The alteration observed in the molecular species of the 10 phospholipid molecular species (selected from 25 most discriminating phospholipid molecular species according to one-way ANOVA and Tukey’s post-hoc tests) in the 3D cultured fibroblasts comparing control (Ctr) with rutin (Rut (25 µM)), control with ascorbic acid (Asc (100 µM)), control with Rut+Asc, UVA with Rut, UVA with UVA+Asc, UVA with UVA+Rut+Asc, UVB with Rut, UVB with UVB+Asc, and UVB with UVB+Rut+Asc, along with their respective fold change. All the alterations are significant at the *p* < 0.05 level. The bold indicates high fold change (more than 2-fold); red arrows indicate the increased level; blue arrows indicate the decreased level; -, not significant changes. The table with the 25 most discriminating phospholipid molecular species is included in Supplementary Materials (Supplementary Table S3).

Phospholipid Species	Log_2_ (Fold-Change)										
	Rut vs. Ctr	Asc vs. Ctr	Rut+Asc vs. Ctr	UVA vs. Ctr	UVB vs. Ctr	UVA+Rut vs. UVA	UVA+Asc vs. UVA	UVA+Rut+Asc vs. UVA	UVB+Rut vs. UVB	UVB+Asc vs. UVB	UVB+Rut +Asc vs. UVB
PCp(44:9)/PCo(44:10)	-	-	1.08 ↑	0.58 ↑	1.00 ↑	-	-	-	1.94 ↑	**2.08** ↑	**2.90** ↑
PCp(44:11)/PCo(44:12)	-	-	**2.13** ↑	1.26 ↑	1.86 ↑	-	-	-	1.88 ↑	1.15 ↑	**2.68** ↑
PC(40:6)	-	-	1.69 ↑	**2.05** ↑	**2.76** ↑	-	-	-	0.70 ↓	0.51 ↑	**2.34** ↑
PC(38:3)	-	-	**2.34** ↑	**2.42** ↑	**3.49** ↑	-	-	-	0.64 ↑	0.53 ↑	1.68 ↑
PS(40:1)	-	-	-	1.31 ↑	**2.15** ↓	-	-	-	-	-	**4.98** ↑
PS(44:4)	-	-	-	1.20 ↑	**2.42** ↓	-	-	-	-	-	**4.93** ↑
SM(d40:1)	1.09 ↓	1.36 ↓	1.88 ↓	**2.25** ↓	1.18 ↓	-	-	**4.46** ↓	**2.85** ↓	0.59 ↓	**3.99** ↓
PI(40:10)	-	-	-	0.57 ↑	0.90 ↓	0.62 ↓	0.66 ↓	1.22 ↑	1.71 ↓	**2.42** ↓	**2.80** ↓
PI(40:3)	-	-	-	1.15 ↓	**2.94** ↓	0.86 ↑	-	**2.76** ↑	0.88 ↓	0.57 ↓	1.34 ↓
PI(40:8)	-	-	-	1.00 ↑	0.93 ↓	0.81 ↓	**2.89** ↓	**2.53** ↑	**2.34** ↓	1.97 ↓	1.95 ↓

**Table 2:** The alteration observed in the molecular species of the 10 ceramide molecular species (selected from 25 most discriminating ceramide molecular species according to one-way ANOVA and Tukey’s post-hoc tests) in the 3D cultured fibroblasts comparing control (Ctr) with rutin (Rut (25 µM)), control with ascorbic acid (Asc (100 µM)), control with Rut+Asc, UVA with Rut, UVA with UVA+Asc, UVA with UVA+Rut+Asc, UVB with Rut, UVB with UVB+Asc, and UVB with UVB+Rut+Asc, along with their respective fold change. All the alterations are significant at the *p* < 0.05 level. Abbreviations: non-hydroxy fatty acid [N], α-hydroxy fatty acid [A], and esterified ω-hydroxy fatty acid [EO], dihydrosphingosine [DS], sphingosine [S], and phytosphingosine [P]. The bold indicates high fold change (more than 2-fold); red arrows indicate the increased level; blue arrows indicate the decreased level; -, not significant changes. The table with the 25 most discriminating ceramide molecular species is included in Supplementary Materials (Supplementary Table S4).

CER Class	Ceramide Species	Log_2_ (Fold-Change)										
		Rut vs. Ctr	Asc vs. Ctr	Rut +Asc vs. Ctr	UVA vs. Ctr	UVB vs. Ctr	UVA +Rut vs. UVA	UVA +Asc vs. UVA	UVA +Rut +Asc vs. UVA	UVB +Rut vs. UVB	UVB +Asc vs. UVB	UVB +Rut +Asc vs. UVB
CER[ADS]	Cer(d18:0/20:0(2OH))	**2.51** ↑	**3.57** ↑	-	-	**3.81** ↑	-	**3.54** ↑	1.85 ↓	**5.40** ↓	0.79 ↑	**2.13** ↑
CER[ADS]	Cer(d18:0/22:0(2OH))	0.48 ↓	1.50 ↓	0.65 ↓	0.31 ↑	**2.72** ↓	-	**3.51** ↓	-	**2.68** ↑	-	**2.22** ↑
CER[AS]	Cer(d16:2/24:0(2OH))	0.75 ↓	1.95 ↓	0.62 ↓	1.17 ↓	-	1.75 ↑	-	0.98 ↓	1.02 ↑	0.28 ↑	0.86 ↑
CER[NP]	Cer(t18:0/22:0)	-	-	-	0.75 ↓	-	0.28 ↓	1.32 ↓	**4.39** ↑	-	-	1.70 ↑
CER[NS]	Cer(d18:2/15:0)	-	-	1.86 ↑	**3.10** ↑	1.15 ↓	**2.77** ↓	**5.36** ↓	1.60 ↓	-	-	**2.84** ↑
CER[NS]	Cer(d18:1/24:0)	-	-	1.79 ↑	1.69 ↑	**2.34** ↓	1.30 ↓	1.13 ↓	1.85 ↑	**3.54** ↑	-	**4.47** ↑
CER[NS]	Cer(d18:1/17:0)	-	-	0.86 ↑	1.13 ↑	-	2.12 ↓	1.47 ↓	**2.82** ↑	**2.37** ↑	-	**2.92** ↑
CER[NDS]	Cer(d18:0/18:0)	-	-	1.79 ↑	0.58 ↓	1.86 ↑	1.99 ↓	0.45 ↑	**4.03** ↑	-	**2.77** ↓	0.32 ↑
CER[NDS]	Cer(d18:0/13:0)	-	**2.39** ↓	0.94 ↑	1.70 ↑	0.79 ↑	**2.71** ↓	**2.29** ↓	**2.27** ↑	1.24 ↑	0.60 ↓	**2.28** ↑
CER[NDS]	Cer(d18:0/20:0)	-	-	-	-	-	-	-	1.97 ↑	1.17 ↑	1.86 ↓	**2.66** ↑

## Data Availability

The data presented in this study are available in supplementary material.

## Supplementary Materials

The following are available online at https://www.mdpi.com/article/10.3390/antiox10040578/s1, Table S1: Phospholipid molecular species identified in the 3D cultured fibroblasts used in the present study. (phosphatidylcholine (PC), lyso-PC (LPC), phosphatidylethanolamine (PE), lyso-PE (LPE), phosphatidylinositols (PI), phosphatidylserine (PS), and sphingomyelin (SM)). Table S2: Ceramide molecular species (63) identified in the 3D cultured fibroblasts used in present study (non-hydroxy fatty acid [N], α-hydroxy fatty acid [A], and esterified ω-hydroxy fatty acid [EO], dihydrosphingosine [DS], sphingosine [S], and phytosphingosine [P]). Table S3: The alteration observed in the molecular species of the 25 most discriminating phospholipid molecular species (according to one-way ANOVA and Tukey’s post-hoc tests) in the 3D cultured fibroblasts comparing control (Ctr) with rutin (Rut (25 µM)), control with ascorbic acid (Asc (100 µM)), control with Rut+Asc, UVA with Rut, UVA with UVA+Asc, UVA with UVA+Rut+Asc, UVB with Rut, UVB with UVB+Asc, and UVB with UVB+Rut+Asc, along with their respective fold change. Table S4: The alteration observed in the molecular species of the 25 most discriminating ceramide molecular species (according to one-way ANOVA and Tukey’s post-hoc tests) in the 3D cultured fibroblasts comparing control (Ctr) with rutin (Rut (25 µM)), control with ascorbic acid (Asc (100 µM)), control with Rut+Asc, UVA with Rut, UVA with UVA+Asc, UVA with UVA+Rut+Asc, UVB with Rut, UVB with UVB+Asc, and UVB with UVB+Rut+Asc, along with their respective fold change. All the alterations are significant at the p < 0.05 level. Abbreviations: non-hydroxy fatty acid [N], α-hydroxy fatty acid [A], and esterified ω-hydroxy fatty acid [EO], dihydrosphingosine [DS], sphingosine [S], and phytosphingosine [P]. The bold indicates high fold change (more than 2-fold); n.s., not significant changes. Table S5: Peak area of each phospholipid molecular species identified in the 3D cultured fibroblasts used in the present study. Data obtained using MZmine software (XLSX). Supplementary Table S6: Peak area of each ceramide molecular species identified in the 3D cultured fibroblasts used in the present study. Data obtained using MZmine software (XLSX).
