# Supplementary material for: Effects of Natural Antioxidants on Phospholipid and Ceramide Profiles of 3D-Cultured Skin Fibroblasts Exposed to UVA or UVB Radiation
Source: Antioxidants (Basel). 2021 Apr 8;10(4):578. doi: 10.3390/antiox10040578 (PMC8068794; doi:10.3390/antiox10040578)
Supplement: Supplementary file 1 [file antioxidants-10-00578-s001.zip › Supplementary Table S3.docx]

**Supplementary Table S3**

The alteration observed in the molecular species of the 25 most discriminating phospholipid molecular species (according to One-way ANOVA and Tukey’s post-hoc tests) in the 3D cultured fibroblasts comparing control (Ctr) with rutin (Rut [25 µM]), control with ascorbic acid (Asc [100 µM]), control with Rut+Asc, UVA with Rut, UVA with UVA +Asc, UVA with UVA+Rut+Asc, UVB with Rut, UVB with UVB +Asc, and UVB with UVB+Rut+Asc, along with their respective fold change. All the alteration are significant at the P < 0.05 level. The bold indicates high fold change (more than 2 fold); n.s., not significant changes.

| **Phospholipid specie** | **Log_2_ (fold-change)** | | | | | | | | | | |
| --- | --- | --- | --- | --- | --- | --- | --- | --- | --- | --- | --- |
|  | Rut vs **Ctr** | Asc vs **Ctr** | Rut+Asc vs **Ctr** | UVA vs **Ctr** | UVB vs **Ctr** | UVA  +Rut vs **UVA** | UVA  +Asc vs **UVA** | UVA  +Rut+  Asc **vs UVA** | UVB  +Rut vs **UVB** | UVB  +Asc vs **UVB** | UVB  +Rut+  Asc vs **UVB** |
| PCp(44:9)/PCo(44:10) | n.s. | n.s. | 1.08 **↑** | 0.58 **↑** | 1.00 **↑** | n.s. | n.s. | n.s. | 1.94 **↑** | **2.08** **↑** | **2.90 ↑** |
| PCp(44:11)/PCo(44:12) | n.s. | n.s. | **2.13** **↑** | 1.26 **↑** | 1.86 **↑** | n.s. | n.s. | n.s. | 1.88 **↑** | 1.15 **↑** | **2.68 ↑** |
| PCp(42:8)/PCo(42:9) | n.s. | n.s. | 1.77 **↑** | 1.49 **↑** | 1.91 **↑** | n.s. | n.s. | n.s. | 1.17 **↑** | 1.93 **↑** | **2.64 ↑** |
| PCp(46:11)/PCo(46:12) | n.s. | n.s. | 1.44 **↑** | 1.59 **↑** | **2.19 ↑** | n.s. | n.s. | n.s. | 0.96 **↑** | 0.77 **↑** | **2.84 ↑** |
| PC(34:0) | n.s. | n.s. | 1.07 **↑** | **2.27 ↑** | **2.90 ↑** | n.s. | n.s. | n.s. | n.s. | 0.80 **↑** | **2.04 ↑** |
| PC(36:1) | n.s. | n.s. | 1.87 **↑** | **2.28 ↑** | **3.15 ↑** | n.s. | n.s. | n.s. | 1.28 **↓** | 0.62 **↑** | 1.98 **↑** |
| PC(40:6) | n.s. | n.s. | 1.69 **↑** | **2.05 ↑** | **2.76 ↑** | n.s. | n.s. | n.s. | 0.70 **↓** | 0.51 **↑** | **2.34 ↑** |
| PC(38:5) | n.s. | n.s. | 1.16 **↑** | 1.41 **↑** | 1.71 **↑** | n.s. | n.s. | n.s. | 0.56 **↓** | 0.69 **↑** | **2.39 ↑** |
| PC(38:3) | n.s. | n.s. | **2.34** **↑** | **2.42 ↑** | **3.49 ↑** | n.s. | n.s. | n.s. | 0.64 **↑** | 0.53 **↑** | 1.68 **↑** |
| PC (40:5) | n.s. | n.s. | 1.76 **↑** | **2.29 ↑** | **3.27 ↑** | n.s. | n.s. | n.s. | 0.55 **↑** | 0.51 **↑** | **2.23 ↑** |
| PS(42:3) | n.s. | n.s. | n.s. | 1.28 **↑** | **2.30 ↓** | n.s. | n.s. | n.s. | n.s. | n.s. | **4.69 ↑** |
| PS(40:3) | n.s. | n.s. | n.s. | 1.23 **↑** | 1.91 **↓** | n.s. | n.s. | n.s. | n.s. | n.s. | **4.34 ↑** |
| PS(42:4) | n.s. | n.s. | n.s. | 1.07 **↑** | **2.43 ↓** | n.s. | n.s. | n.s. | n.s. | n.s. | **4.46 ↑** |
| PS(40:1) | n.s. | n.s. | n.s. | 1.31 **↑** | **2.15 ↓** | n.s. | n.s. | n.s. | n.s. | n.s. | **4.98 ↑** |
| PS(38:1) | n.s. | n.s. | n.s. | 1.67 **↑** | **2.79 ↓** | n.s. | n.s. | n.s. | n.s. | n.s. | **4.71 ↑** |
| PS(38:0) | n.s. | n.s. | n.s. | 0.51 **↑** | **2.63 ↓** | n.s. | n.s. | n.s. | n.s. | n.s. | **4.29 ↑** |
| PS(42:2) | n.s. | n.s. | n.s. | 1.06 **↑** | **2.48 ↓** | n.s. | n.s. | n.s. | n.s. | n.s. | **4.65 ↑** |
| PS(44:4) | n.s. | n.s. | n.s. | 1.20 **↑** | **2.42 ↓** | n.s. | n.s. | n.s. | n.s. | n.s. | **4.93 ↑** |
| SM(d40:1) | 1.09 **↓** | 1.36 **↓** | 1.88 **↓** | **2.25** **↓** | 1.18 **↓** | n.s. | n.s. | **4.46** **↓** | **2.85** **↓** | 0.59 **↓** | **3.99** **↓** |
| PI(40:4) | n.s. | n.s. | n.s. | 0.91 **↑** | 0.49 **↓** | n.s. | n.s. | n.s. | 1.91 **↓** | **2.90 ↓** | **3.63 ↓** |
| PI(40:10) | n.s. | n.s. | n.s. | 0.57 **↑** | 0.90 **↓** | 0.62 ↓ | 0.66 **↓** | 1.22 **↑** | 1.71 **↓** | **2.42 ↓** | **2.80 ↓** |
| PI(40:2) | n.s. | n.s. | n.s. | 1.43 **↑** | 0.62 **↓** | 0.98 ↓ | 1.63 **↓** | n.s. | 1.65 **↓** | 1.64 **↓** | 1.88 **↓** |
| PI(42:6) | n.s. | n.s. | n.s. | 1.76 **↑** | 0.46 **↓** | 1.17 ↓ | 1.99 **↓** | n.s. | 1.82 **↓** | **2.86** **↓** | **2.60** **↓** |
| PI(40:3) | n.s. | n.s. | n.s. | 1.15 **↓** | **2.94** **↓** | 0.86 ↑ | n.s. | **2.76 ↑** | 0.88 **↓** | 0.57 **↓** | 1.34 **↓** |
| PI(40:8) | n.s. | n.s. | n.s. | 1.00 **↑** | 0.93 **↓** | 0.81 ↓ | **2.89** **↓** | **2.53 ↑** | **2.34** **↓** | 1.97 **↓** | 1.95 **↓** |
