# Supplementary material for: Effects of Natural Antioxidants on Phospholipid and Ceramide Profiles of 3D-Cultured Skin Fibroblasts Exposed to UVA or UVB Radiation
Source: Antioxidants (Basel). 2021 Apr 8;10(4):578. doi: 10.3390/antiox10040578 (PMC8068794; doi:10.3390/antiox10040578)
Supplement: Supplementary file 1 [file antioxidants-10-00578-s001.zip › Supplementary Table S4.docx]

**Supplementary Table S4.**

The alteration observed in the molecular species of the 25 most discriminating ceramide molecular species (according to One-way ANOVA and Tukey’s post-hoc tests) in the 3D cultured fibroblasts comparing control (Ctr) with rutin (Rut [25 µM]), control with ascorbic acid (Asc [100 µM]), control with Rut+Asc, UVA with Rut, UVA with UVA +Asc, UVA with UVA+Rut+Asc, UVB with Rut, UVB with UVB +Asc, and UVB with UVB+Rut+Asc, along with their respective fold change. All the alteration are significant at the P < 0.05 level. Abbreviations: non-hydroxy fatty acid [N], α-hydroxy fatty acid [A], and esterified ω-hydroxy fatty acid [EO], dihydrosphingosine [DS], sphingosine [S], and phytosphingosine [P]. The bold indicates high fold change (more than 2 fold); n.s., not significant changes.

| **CER class** | **Ceramide specie** | **Log_2_ (fold-change)** | | | | | | | | | | |
| --- | --- | --- | --- | --- | --- | --- | --- | --- | --- | --- | --- | --- |
|  |  | Rut vs **Ctr** | Asc vs **Ctr** | Rut+  Asc vs **Ctr** | UVA vs **c Ctr** | UVB vs **Ctr** | UVA+Rut vs **UVA** | UVA+Asc vs **UVA** | UVA+Rut+  Asc **vs UVA** | UVB+Rut vs **UVB** | UVB+Asc vs **UVB** | UVB+Rut+  Asc vs **UVB** |
| CER[ADS] | Cer(d18:0/20:0(2OH)) | **2.51** **↑** | **3.57** **↑** | n.s. | n.s. | **3.81** **↑** | n.s. | **3.54 ↑** | 1.85 **↓** | **5.40** **↓** | 0.79 **↑** | **2.13 ↑** |
| CER[ADS] | Cer(d18:0/22:0(2OH)) | 0.48 **↓** | 1.50 **↓** | 0.65 **↓** | 0.31 **↑** | **2.72** **↓** | n.s. | **3.51 ↓** | n.s. | **2.68** **↑** | n.s. | **2.22 ↑** |
| CER[AS] | Cer(d16:2/24:0(2OH)) | 0.75 **↓** | 1.95 **↓** | 0.62 **↓** | 1.17 **↓** | n.s. | 1.75 **↑** | n.s. | 0.98 **↓** | 1.02 **↑** | 0.28 **↑** | 0.86 **↑** |
| CER[NS] | Cer(d18:2/15:0) | n.s. | n.s. | 1.86 **↑** | **3.10** **↑** | 1.15 **↓** | **2.77** **↓** | **5.36 ↓** | 1.60 **↓** | n.s. | n.s. | **2.84 ↑** |
| CER[NS] | Cer(d18:1/24:0) | n.s. | n.s. | 1.79 **↑** | 1.69 **↑** | **2.34 ↓** | 1.30 **↓** | 1.13 **↓** | 1.85 **↑** | **3.54** **↑** | n.s. | **4.47 ↑** |
| CER[NS] | Cer(d16:1/23:0) | n.s. | n.s. | 1.97 **↑** | 1.76 **↑** | **3.13 ↓** | **2.07** **↓** | 1.29 **↓** | **3.03 ↑** | 1.14 **↑** | n.s. | **2.43 ↑** |
| CER[NS] | Cer(d18:2/21:0) | 1.74 **↓** | n.s. | 0.90 **↓** | 1.25 **↑** | n.s. | **2.61** **↓** | 1.78 **↓** | **2.06 ↑** | **2.00** **↑** | 1.44 **↓** | **n.s.** |
| CER[NS] | Cer(d18:0/17:0) | n.s. | n.s. | 1.30 **↑** | 1.55 **↑** | 0.62 **↓** | **4.14 ↓** | 0.45 **↓** | 1.09 **↑** | **2.03 ↑** | 1.02 **↓** | 1.79 **↑** |
| CER[NS] | Cer(d18:1/18:0) | n.s. | 1.27 **↓** | 1.32 **↑** | 1.16 **↑** | 1.81 **↑** | **2.52 ↓** | **2.04 ↓** | **2.38 ↑** | 1.23 **↑** | n.s. | 0.32 **↑** |
| CER[NS] | Cer(d18:1/22:0) | n.s. | 1.46 **↓** | 0.96 **↑** | 1.05 **↑** | 1.68 **↑** | 1.52 ↓ | 1.78 **↓** | **3.73 ↑** | 0.92 **↑** | 1.18 **↓** | 1.74 **↑** |
| CER[NS] | Cer(d16:1/17:0) | n.s. | 1.90 **↓** | 1.88 **↑** | **2.49 ↑** | 1.51 **↑** | **2.53 ↓** | **2.65 ↓** | 1.96 **↑** | 1.57 **↑** | 0.77 **↓** | 1.78 **↑** |
| CER[NS] | Cer(d18:1/25:0) | **2.95** **↑** | n.s. | n.s. | **2.98 ↑** | **2.98 ↑** | 1.38 **↓** | **2.09 ↓** | **3.53** **↑** | 1.96 **↑** | 0.38 **↓** | 1.82 **↑** |
| CER[NS] | Cer(d18:2/20:0) | n.s. | n.s. | 1.44 **↑** | 1.43 **↑** | n.s. | **2.19 ↓** | 0.93 **↓** | **2.06** **↑** | **2.36** **↑** | 0.45 **↓** | 1.47 **↑** |
| CER[NS] | Cer(d18:2/22:0) | n.s. | n.s. | n.s. | **2.11** **↑** | n.s. | **2.53 ↓** | 1.99 **↓** | 1.92 **↑** | 0.92 **↑** | 0.55 **↓** | **2.97 ↑** |
| CER[NS] | Cer(d18:1/19:0) | n.s. | n.s. | 1.25 **↑** | 1.27 **↑** | n.s. | 1.91 **↓** | 1.46 **↓** | **3.01 ↑** | **2.25 ↑** | n.s. | **2.38 ↑** |
| CER[NS] | Cer(d18:1/17:0) | n.s. | n.s. | 0.86 **↑** | 1.13 **↑** | n.s. | 2.12 **↓** | 1.47 **↓** | **2.82 ↑** | **2.37 ↑** | n.s. | **2.92 ↑** |
| CER[NDS] | Cer(d18:0/18:0) | n.s. | n.s. | 1.79 **↑** | 0.58 **↓** | 1.86 **↑** | 1.99 **↓** | 0.45 **↑** | **4.03 ↑** | n.s. | **2.77** **↓** | 0.32 **↑** |
| CER[NDS] | Cer(d18:0/15:0) | n.s. | 1.67 **↓** | 1.25 **↑** | 1.78 **↑** | n.s. | **2.78 ↓** | 0.68 **↓** | 0.61 **↓** | n.s. | **2.80** **↓** | 1.68 **↑** |
| CER[NDS] | Cer(d18:0/26:0) | 1.98 **↓** | **2.68** **↓** | n.s. | **2.01 ↑** | 1.72 **↑** | 1.92 **↓** | **2.50 ↓** | **3.03 ↑** | 1.65 **↑** | 0.72 **↓** | **2.57** **↑** |
| CER[NDS] | Cer(d18:0/13:0) | n.s. | **2.39** **↓** | 0.94 **↑** | 1.70 **↑** | 0.79 **↑** | **2.71 ↓** | **2.29 ↓** | **2.27 ↑** | 1.24 **↑** | 0.60 **↓** | **2.28 ↑** |
| CER[NDS] | Cer(d20:0/26:0) | n.s. | n.s. | n.s. | **3.04 ↑** | **3.95 ↑** | **4.36 ↓** | **2.40 ↓** | 1.37 **↑** | 1.59 **↑** | n.s. | **5.15 ↑** |
| CER[NDS] | Cer(d18:0/16:0) | n.s. | n.s. | n.s. | 1.60 **↑** | 0.39 **↑** | **3.83 ↓** | **2.39 ↓** | 1.97 **↑** | 1.58 **↑** | n.s. | **2.89 ↑** |
| CER[NDS] | Cer(d18:0/14:0) | 1.29 **↓** | n.s. | n.s. | **2.39** **↑** | 1.27 **↑** | **3.82 ↓** | **2.24 ↓** | 1.53 **↑** | 0.61 **↑** | 0.99 **↓** | **2.19 ↑** |
| CER[NDS] | Cer(d18:0/20:0) | n.s. | n.s. | n.s. | n.s. | n.s. | n.s. | n.s. | 1.97 **↑** | 1.17 **↑** | 1.86 **↓** | **2.66 ↑** |
| CER[NP] | Cer(t18:0/22:0) | n.s. | n.s. | n.s. | 0.75 **↓** | n.s. | 0.28 **↓** | 1.32 **↓** | **4.39 ↑** | n.s. | n.s. | 1.70 **↑** |
