# Supplementary material for: Effects of Natural Antioxidants on Phospholipid and Ceramide Profiles of 3D-Cultured Skin Fibroblasts Exposed to UVA or UVB Radiation
Source: Antioxidants (Basel). 2021 Apr 8;10(4):578. doi: 10.3390/antiox10040578 (PMC8068794; doi:10.3390/antiox10040578)
Supplement: Supplementary file 1 [file antioxidants-10-00578-s001.zip › Supplementary materials S1- S2.docx]

**Supplementary materials**

**Supplementary Table S1.** Phospholipid molecular species identified in the 3D cultured fibroblasts used in the present study. *(*phosphatidylcholine (PC), lyso-PC (LPC), phosphatidylethanolamine (PE), lyso-PE (LPE), phosphatidylinositols (PI), phosphatidylserine (PS), and sphingomyelin (SM)).

| **Phospholipid class** | **m/z** | **Retention time** | **Phospholipid specie** |
| --- | --- | --- | --- |
| **PC** | 868.605 | 16.89 | PC(38:4) |
|  | 840.5744 | 17.24 | PC(36:4) |
|  | 818.5891 | 17.96 | PC(34:1) |
|  | 816.5765 | 17.99 | PC(34:2) |
|  | 844.6037 | 17.59 | PC(36:2) |
|  | 866.5916 | 16.97 | PC(38:5) |
|  | 868.5488 | 16.87 | PCp(40:10) |
|  | 864.5751 | 16.97 | PC(38:6) |
|  | 846.6238 | 17.59 | PC(36:1) |
|  | 892.6062 | 16.73 | PC(40:6) |
|  | 842.5901 | 17.51 | PC(36:3) |
|  | 870.6218 | 16.94 | PC(38:3) |
|  | 820.608 | 18.05 | PC(34:0) |
|  | 792.577 | 18.29 | PC(32:0) |
|  | 950.6241 | 16.70 | PCp(46:11)/PCo(46:12) |
|  | 790.5605 | 18.25 | PC(32:1) |
|  | 894.6218 | 16.75 | PC(40:5) |
|  | 844.5498 | 17.37 | PCp(38:8) |
|  | 870.5663 | 16.96 | PCp(40:9)/PCo(40:10) |
|  | 922.5899 | 17.12 | PCp(44:11)/PCo(44:12) |
|  | 898.5905 | 18.05 | PCp(42:9)/PCo(42:10) |
|  | 900.6042 | 17.99 | PCp(42:8)/PCo(42:9) |
|  | 926.6201 | 17.70 | PCp(44:9)/PCo(44:10) |
| **LPC** | 554.3429 | 22.46 | LPC(16:0) |
|  | 578.3439 | 22.39 | LPC(18:2) |
|  | 582.3799 | 22.03 | LPC(18:0) |
|  | 580.3616 | 22.40 | LPC(18:1) |
|  | 602.3452 | 21.92 | LPC(20:4) |
|  | 552.3289 | 23.44 | LPC(16:1) |
| **PE** | 766.538 | 7.35 | PE(38:4) |
|  | 714.5093 | 8.08 | PE(34:2) |
|  | 750.5427 | 7.36 | PEo(38:5)/PEp(38:4) |
|  | 738.5123 | 7.52 | PE(36:4) |
|  | 744.5529 | 7.72 | PE(36:1) |
|  | 742.5388 | 7.72 | PE(36:2) |
|  | 702.5444 | 7.93 | PEo(34:1)/PEp(34:0) |
|  | 764.5231 | 7.41 | PE(38:5) |
|  | 762.5082 | 7.39 | PE(38:6) |
|  | 752.5569 | 7.50 | PEo(38:4)/PEp(38:3) |
|  | 716.5206 | 7.84 | PE(34:1) |
|  | 748.5252 | 7.35 | PEo(38:6)/PEp(38:5) |
|  | 674.5125 | 7.96 | PEo(32:1)/PEp(32:0) |
|  | 722.5125 | 7.47 | PEo(36:5)/PEp(36:4) |
|  | 790.5409 | 7.24 | PE(40:6) |
|  | 766.4835 | 7.34 | PEp(40:10) |
|  | 740.5218 | 7.63 | PE(36:3) |
|  | 700.5274 | 7.92 | PEo(34:2)/PEp(34:1) |
|  | 768.5506 | 7.40 | PE(38:3) |
|  | 774.5416 | 7.24 | PEo(40:7)/PEp(40:6) |
|  | 776.5591 | 7.37 | PEo(40:6)/PEp(40:5) |
|  | 778.5742 | 7.43 | PEo(40:5)/PEp(40:4) |
|  | 728.5578 | 7.75 | PEo(36:2)/PEp(36:1) |
|  | 792.5519 | 7.32 | PE(40:5) |
|  | 726.541 | 7.96 | PEo(36:3)/PEp(36:2) |
|  | 724.5254 | 7.82 | PEo(36:4)/PEp(36:3) |
|  | 742.4819 | 7.87 | PEp(38:8) |
|  | 788.5226 | 7.74 | PE(40:7) |
|  | 780.5871 | 7.51 | PEo(40:4)/PEp(40:3) |
|  | 794.5689 | 7.47 | PE(40:4) |
|  | 746.5114 | 7.48 | PEo(38:7)/PEp(38:6) |
|  | 768.4974 | 7.52 | PEo(40:10)/PEp(40:9) |
|  | 634.4447 | 8.16 | PE(28:0) |
|  | 736.4931 | 7.62 | PE(36:5) |
| **LPE** | 500.2809 | 10.01 | LPE(20:4) |
|  | 480.3087 | 10.24 | LPE(18:0) |
|  | 452.2792 | 10.53 | LPE(16:0) |
|  | 478.2954 | 10.40 | LPE(18:1) |
|  | 476.2781 | 10.54 | LPE(18:2) |
|  | 524.2809 | 9.96 | LPE(22:6) |
|  | 526.2905 | 9.97 | LPE(22:5) |
|  | 502.2911 | 10.64 | LPE(20:3) |
| **PI** | 885.5493 | 4.47 | PI(38:4) |
|  | 913.5769 | 3.68 | PI(40:4) |
|  | 943.6299 | 3.63 | PI(42:3) |
|  | 915.5964 | 3.92 | PI(40:3) |
|  | 779.4677 | 3.92 | PI(30:1) |
|  | 857.5163 | 4.41 | PI(36:4) |
|  | 945.6497 | 3.63 | PI(42:2) |
|  | 877.4893 | 3.67 | PI(38:8) |
|  | 859.5323 | 4.74 | PI(36:3) |
|  | 833.5189 | 4.75 | PI(34:2) |
|  | 883.5344 | 4.53 | PI(38:5) |
|  | 901.4818 | 4.40 | PI(40:10) |
|  | 917.6087 | 4.17 | PI(40:2) |
|  | 911.5631 | 4.65 | PI(40:5) |
|  | 937.5817 | 4.46 | PI(42:6) |
|  | 905.518 | 3.93 | PI(40:8) |
|  | 879.503 | 3.67 | PI(38:7) |
|  | 868.605 | 16.89 | PS(42:3) |
| **PS** | 840.5744 | 17.24 | PS(40:3) |
|  | 816.5765 | 17.96 | PS(38:1) |
|  | 844.6037 | 17.59 | PS(40:1) |
|  | 818.5891 | 17.94 | PS(38:0) |
|  | 866.5916 | 16.97 | PS(42:4) |
|  | 864.5751 | 16.97 | PS(42:5) |
|  | 870.6218 | 16.94 | PS(42:2) |
|  | 892.6062 | 16.73 | PS(44:5) |
|  | 842.5901 | 17.51 | PS(40:2) |
|  | 846.6238 | 17.59 | PS(40:0) |
|  | 894.6218 | 16.75 | PS(44:4) |
|  | 790.5605 | 18.25 | PS(36:0) |
| **SM** | 761.5823 | 20.35 | SM(d34:1) |
|  | 871.6902 | 19.36 | SM(d42:2) |
|  | 845.6743 | 19.54 | SM(d40:1) |

**Supplementary Table S2.** Ceramide molecular species (63) identified in the 3D cultured fibroblasts used in present study *(non-hydroxy fatty acid [N], α-hydroxy fatty acid [A], and esterified ω-hydroxy fatty acid [EO], dihydrosphingosine [DS], sphingosine [S], and phytosphingosine [P]).*

| **CER class** | **CER specie** | | |
| --- | --- | --- | --- |
|  | **m/z** | **RT** | **Name** |
| **CER[EOS]** | 748.7123 | 32.35 | 1-O-myristoyl-Cer(d18:1/16:0) |
|  | 776.7407 | 35.88 | 1-O-palmitoyl-Cer(d18:1/16:0) |
|  | 832.8001 | 35.60 | 1-O-eicosanoyl-Cer(d18:1/16:0) |
| **CER[AS]** | 580.5217 | 40.72 | Cer(d18:2/18:0(2OH)) |
|  | 552.4928 | 36.45 | Cer(d16:1/18:1(2OH)) |
|  | 610.5688 | 34.28 | Cer(d18:1/20:0(2OH)) |
|  | 636.587 | 42.70 | Cer(d16:2/24:0(2OH)) |
|  | 638.6001 | 38.73 | Cer(d16:1/24:0(2OH)) |
|  | 578.508 | 37.47 | Cer(d16:2/20:1(2OH)) |
|  | 606.5384 | 41.60 | Cer(d16:2/22:1(2OH)) |
|  | 594.5367 | 37.56 | Cer(d18:2/19:0(2OH)) |
|  | 566.5072 | 35.76 | Cer(d15:2/20:0(2OH)) |
|  | 554.5058 | 36.45 | Cer(d18:1/16:0(2OH)) |
|  | 608.5533 | 42.49 | Cer(d18:2/20:0(2OH)) |
|  | 524.4628 | 32.19 | Cer(d14:1/18:1(2OH)) |
|  | 550.4753 | 36.63 | Cer(d16:2/18:1(2OH)) |
|  | 582.5389 | 33.81 | Cer(d16:1/20:0(2OH)) |
| **CER[NS]** | 648.6221 | 41.35 | Cer(d18:1/24:1) |
|  | 580.5586 | 40.72 | Cer(d18:1/19:0) |
|  | 622.6061 | 40.82 | Cer(d18:1/22:0) |
|  | 650.6378 | 44.15 | Cer(d18:1/24:0) |
|  | 636.6221 | 42.71 | Cer(d18:1/23:0) |
|  | 552.5255 | 36.44 | Cer(d18:1/17:0) |
|  | 634.6053 | 34.59 | Cer(d18:2/23:0) |
|  | 620.5902 | 40.64 | Cer(d18:2/22:0) |
|  | 594.5756 | 37.25 | Cer(d18:1/20:0) |
|  | 592.5588 | 36.45 | Cer(d18:2/20:0) |
|  | 608.5917 | 42.85 | Cer(d16:1/23:0) |
|  | 566.5447 | 34.14 | Cer(d18:1/18:0) |
|  | 606.577 | 41.26 | Cer(d18:2/21:0) |
|  | 676.6535 | 44.14 | Cer(d18:1/26:1) |
|  | 538.5124 | 32.72 | Cer(d18:1/16:0) |
|  | 664.6494 | 33.68 | Cer(d18:1/25:0) |
|  | 524.4961 | 32.15 | Cer(d16:1/17:0) |
|  | 590.5455 | 34.41 | Cer(d18:2/20:1) |
|  | 678.6674 | 47.28 | Cer(d18:1/26:0) |
|  | 522.4827 | 44.01 | Cer(d18:2/15:0) |
|  | 564.5265 | 32.82 | Cer(d18:1/18:1) |
|  | 510.4838 | 43.76 | Cer(d18:1/14:0) |
| **CER[NDS]** | 498.4824 | 39.67 | Cer(d18:0/13:0) |
|  | 554.5433 | 41.84 | Cer(d18:0/17:0) |
|  | 568.5583 | 34.55 | Cer(d18:0/18:0) |
|  | 512.4976 | 27.74 | Cer(d18:0/14:0) |
|  | 680.6857 | 30.33 | Cer(d18:0/26:0) |
|  | 566.5447 | 33.55 | Cer(d18:0/18:1) |
|  | 596.5874 | 39.78 | Cer(d18:0/20:0) |
|  | 708.7123 | 24.63 | Cer(d20:0/26:0) |
|  | 540.5286 | 33.20 | Cer(d18:0/16:0) |
|  | 526.5134 | 38.67 | Cer(d18:0/15:0) |
| **CER[ADS]** | 612.5811 | 35.67 | Cer(d18:0/20:0(2OH)) |
|  | 668.6441 | 28.24 | Cer(d18:0/24:0(2OH)) |
|  | 640.6129 | 38.79 | Cer(d18:0/22:0(2OH)) |
|  | 556.5225 | 25.75 | Cer(d18:0/16:0(2OH)) |
|  | 584.5554 | 30.94 | Cer(d18:0/18:0(2OH)) |
|  | 696.6778 | 32.56 | Cer(d18:0/26:0(2OH)) |
| **CER[NP]** | 640.6156 | 40.39 | Cer(t18:0/22:0) |
|  | 668.6455 | 40.16 | Cer(t18:0/24:0) |
| **CER[AP]** | 598.5344 | 40.73 | Cer(t26:1/10:0(2OH[R])) |
|  | 600.5481 | 38.61 | Cer(t18:0/18:0(2OH)) |
|  | 628.5774 | 42.45 | Cer(t18:0/20:0(2OH)) |
|  | 684.6421 | 42.93 | Cer(t18:0/24:0(2OH)) |
|  | 626.5653 | 43.26 | Cer(t18:1/20:0(2OH)) |
|  | 656.6152 | 42.18 | Cer(t18:0/22:0(2OH)) |
